# Evaluation of 2,3-Butanediol Production from Red Seaweed *Gelidium amansii* Hydrolysates Using Engineered *Saccharomyces cerevisiae*

**DOI:** 10.4014/jmb.2007.07037

**Published:** 2020-09-22

**Authors:** Chae Hun Ra, Jin-Ho Seo, Gwi-Taek Jeong, Sung-Koo Kim

**Affiliations:** 1 Department of Food Science and Biotechnology, College of Engineering, Global K-Food Research Center, Hankyong National University, Anseong 7579, Republic of Korea; 2 Department of Agricultural Biotechnology and Center for Food and Bioconvergence, Seoul National University, Seoul 0886, Republic of Korea; 3 Department of Biotechnology, Pukyong National University, Busan 4851, Republic of Korea

**Keywords:** Hyper-thermal acid hydrolysis, enzymatic saccharification, *Gelidium amansii*, 2, 3-butanediol, engineered *Saccharomyces cerevisiae*

## Abstract

Hyper-thermal (HT) acid hydrolysis of red seaweed *Gelidium amansii* was performed using 12% (w/v) slurry and an acid mix concentration of 180 mM at 150°C for 10 min. Enzymatic saccharification when using a combination of Celluclast 1.5 L and CTec2 at a dose of 16 U/ml led to the production of 12.0 g/l of reducing sugar with an efficiency of enzymatic saccharification of 13.2%. After the enzymatic saccharification, 2,3-butanediol (2,3-BD) fermentation was carried out using an engineered *S. cerevisiae* strain. The use of HT acid-hydrolyzed medium with 1.9 g/l of 5-hydroxymethylfurfural showed a reduction in the lag time from 48 to 24 h. The 2,3-BD concentration and yield coefficient at 72 h were 14.8 g/l and 0.30, respectively. Therefore, HT acid hydrolysis and the use of the engineered *S. cerevisiae* strain can enhance the overall 2,3-BD yields from *G. amansii* seaweed.

## Introduction

An increase in public awareness in regard to renewable energy and climate change has led to a growing interest in the use of microbial fermentation technologies for the production of biofuels and chemicals [1, 2]. The microbial production of 2,3-butanediol (2,3-BD) is one such example, as 2,3-BD has a large number of industrial applications; for example, it can be used as an antifreeze agent due to a low freezing point of –60°C [3]. Moreover, the production of 2,3-BD via microbial fermentation can alleviate the dependence on oil supplies for the production of platform chemicals [4]. Additionally, 2,3-BD has other potential applications, such as the manufacturing of printing inks, perfumes, moistening and softening agents, explosives, plasticizers, foods, and pharmaceuticals [5].

Marine biomass, such as algae and seaweed, can be a potential resource for the biochemical production of biofuel, as it does not contain lignin and has a high carbohydrate content and a fast growth rate, as well as having the ability to fix large amounts of carbon dioxide [6]. In particular, up to 58% of the dry weight of the red algae *Gelidium amansii* is composed of carbohydrates such as agar (galactan) and cellulose. Agar is composed of galactose and 3,6-anhydrogalactose (AHG). Thus, the *G. amansii* hydrolysate, which contains a high amount of glucose and galactose, can be used as a substrate for the microbial production of 2,3-BD.

In this study, we used hyper-thermal (HT) acid hydrolysis to yield high concentrations of monosaccharides and low concentrations of inhibitory compounds. Moreover, several factors that influence hydrolysis efficiency were evaluated, including acid types and concentrations, temperature, thermal hydrolysis time, and slurry concentration. Additionally, we used enzymatic saccharification to hydrolyze the remaining cellulose for glucose production.

Until now, *Klebsiella pneumoniae*, *Enterobacter aerogenes*, *Bacillus* sp. and *Serratia marcescens* have been considered promising for the efficient production of 2,3-BD. However, *Saccharomyces cerevisiae* has many advantages over bacteria for industrial use and significant research efforts have focused on the production of 2,3-BD by engineered *S. cerevisiae* harboring the bacterial 2,3-BD biosynthetic enzymes [7, 8]. The pyruvate decarboxylase (Pdc)-deficient *S. cerevisiae* strain provides a promising metabolic background for the production of non-ethanol products, such as 2,3-BD [7], 3-hydroxypropionic acid [9], and lactic acid [10]. This strain accumulates pyruvate, which is a precursor of numerous chemical molecules, instead of producing ethanol from glucose [11]. Therefore, in this study, the evolved Pdc-deficient *S. cerevisiae* strain expressing the 2,3-BD synthetic enzymes was evaluated to investigate its 2,3-BD production capacity using *G. amansii* hydrolysate.

## Materials and Methods

### Microbial Strains and Culture Medium

The evolved 2,3-BD-producing *S. cerevisiae* strain used in this study was obtained from the Agricultural Biotechnology and Center for Food and Bioconvergence (Seoul National University, Seoul, Republic of Korea). An engineered *S. cerevisiae* strain (BD4) capable of efficient production of 2,3-BD was constructed through the elimination of the pyruvate decarboxylase genes (*ΔPDC1* and *ΔPDC5*), overexpression of acetolactate synthase (*alsS*) and acetolactate decarboxylase (*alsD*), and overexpression of 2,3-butanediol dehydrogenase (*BDH1*) [7]. The seed cultures were prepared according to procedures described by Kim *et al*. and Choi *et al*. [7, 12]. Yeast cells were cultured at 30°C and 150 rpm for 24 h in pre-culture yeast synthetic complete (YSC) medium containing 6.7 g/l yeast nitrogen base (YNB), 20 g/l glucose, and 1 g/l ethanol.

The growth medium for the Pdc-deficient strains was supplemented with two carbon (C_2_) compounds, such as acetate or ethanol, in order to enable the synthesis of lysine and fatty acids [11, 13]. After being cultured for 24 h, the cells in the mid-exponential growth phase were used for the main culture of the yeast strains.

*Gelidium amansii* (product of Morocco) was obtained from Biolsystems Co., Ltd. (Republic of Korea). The composition of *G. amansii* was analyzed by the Feed and Foods Nutrition Research Center of Pukyong National University (Republic of Korea) according to the method provided by the Association of Official Analytical Chemists (AOAC) [14]. Thus, the amounts of carbohydrates and cellulose were used to calculate the efficiency of the pretreatment and enzymatic saccharification.

### Hyper-Thermal Acid Hydrolysis Pretreatment

One-factor-at-a-time (OFAT) experiments were carried out to determine the settings for the indicated main factors [15]. HT acid hydrolysis was performed by changing the pretreatment parameters, such as the H_2_SO_4_, H_3_PO_4_, HCl, and HNO_3_ concentrations, to between 45–720 mM. Meanwhile, temperatures were in the range of 120–180°C, thermal hydrolysis times between 5–20 min, and seaweed slurry concentrations between 8–20% (w/v).

The HT acid hydrolysis pretreatment was initiated by raising the reactor temperature in an oil bath. The reaction was performed using a 50-ml stainless steel batch reactor with a working volume of 40 ml. The stainless steel reactor was filled with the indicated amounts of seaweed slurry and acids. A magnetic stirrer was placed inside the reactor to maintain efficient contact between the seaweed slurry and the acids. The temperature was monitored and adjusted with a proportional-integral-derivative (PID) temperature controller (TC_2_00P; Misung Scientific Co., Ltd., Republic of Korea). After pretreatment, the reactor was quickly cooled in cold water. The HT acid hydrolysis pretreatment efficiency (*E*_p_, %) was calculated using Eq. (1):

(1) $$E_{p}(\%)=\frac{{\mathrm{\Delta S}}_{\mathrm{RS}}}{\mathrm{TC}}\times 100($$

where ΔS_RS_ is the increase in reducing sugar concentration (g/l) during HT acid hydrolysis and TC is the total carbohydrate (g/l) content of carbohydrates and cellulose in the pretreated *G. amansii*.

### Enzymatic Saccharification

After HT acid hydrolysis, the pH of the pretreated *G. amansii* mixture was adjusted to 5.0 with 5 N NaOH. Enzymatic saccharification of the *G. amansii* hydrolysate was carried out using Viscozyme L (β-glucanase, 121 U/ml; Novozymes, Denmark), Celluclast 1.5 L (cellulase, 854 U/ml; Novozymes), and Cellic CTec2 (cellulase, 150 U/ml; Novozymes). The three enzymes used in this study are blended enzymes containing cellulase, β-glucanase (endo-1,3 or 1,4), and hemicellulose, as described in previous reports [16, 17]. The three enzymes were then diluted and an enzyme concentration of 16 U/ml was used. The reaction was performed at 50°C and 150 rpm for 48 h after HT acid hydrolysis. To achieve a synergistic effect compared with single enzyme treatments, the mixed enzymes were prepared at a 1:1 ratio with 16 U/ml of each enzyme. The efficiency of enzymatic saccharification (Es, %) process was determined using Eq. (2).

(2) $$E_{s}(\%)=\frac{{\mathrm{\Delta S}}_{\mathrm{RS}}}{\mathrm{TC}}\times 100$$

where ΔS_RS_ represents the increase in reducing sugar concentration (g/l) during the enzymatic saccharification process and TC represents the concentration of total carbohydrates (g/l), namely carbohydrates and cellulose, in the pretreated *G. amansii*.

### Fermentation of 2,3-Butanediol

After the enzymatic saccharification process, 2,3-BD fermentation was carried out using 100 ml of the *G. amansii* hydrolysate in a 250-ml Erlenmeyer flask under semi-anaerobic conditions. Strictly anaerobic fermentation is normally carried out in a sealed, airtight fermenter or by gas packing with N_2_ gas. Thus, the term ‘semi-anaerobic conditions’ is applied due to the use of a non-airtight container. The *G. amansii* hydrolysates were supplemented with the following nutrients: 2.5 g/l NH_4_Cl, 5.0 g/l K_2_HPO_4_, 0.25 g/l MgSO_4_, and 3.0 g/l yeast extract. The pH of the hydrolysate medium used for fermentation was adjusted to 6.3 by adding 5 N NaOH before the inoculum was added. Ten mL of the engineered *S. cerevisiae* inoculum (6.8 g dcw/L) was transferred to the *G. amansii* hydrolysate and then the fermentation process was carried out at 30°C and 150 rpm. Samples were taken periodically and stored at –20°C to determine the 2,3-BD, reducing sugar, glycerol, and 5-hydroxymethylfurfural (5-HMF) concentrations, as well as the pH and optical density. The 2,3-BD yield (*Y*_BD_, g/g) was determined according to the following Eq. (3).

(3) $$Y_{\mathrm{BD}}(\%)=\frac{{\left[2,3-\mathrm{BD}\right]}_{\max}}{{\left[\mathrm{Reducing}\mathrm{sugar}\right]}_{\mathrm{ini}}}\times 100$$

where [2,3-BD]_max_ is the highest 2,3-BD concentration (g/l) obtained during the fermentation process and [Reducing sugar]_ini_ is the total initial reducing sugar concentration (g/l) at the start of the fermentation process.

### Analytical Methods

The cell concentration was determined based on the correlation between the absorbance at 600 nm (Ultrospec 6300 Pro; Biochrom Ltd., UK) and the dry cell weight (g dcw/L). The reducing sugar concentration was determined using the 3,5-dinitrosalicylic acid (DNS) method with glucose (Sigma-Aldrich, USA) as the standard. The concentrations of 2,3-BD, glycerol, and 5-HMF were determined via high-performance liquid chromatography (HPLC, Agilent 1100 Series; Agilent Technologies, USA) using an Aminex HPX-87H column (Bio-Rad, USA). The detector was used to measure the refractive index. The mobile phase consisted of 5 mM H_2_SO_4_ at a flow rate of 0.6 ml/min at 65°C. The values were reported as the means of triplicate experiments.

## Results and Discussion

### Composition and Pretreatment of *G. amansii*

The *G. amansii* samples were composed of 58.4% carbohydrates, 17.4% crude fiber, 18.7% crude protein, 0.7%crude lipids, and 4.8% crude ash. The total carbohydrate content of the *G. amansii* samples used in this study was 75.8%, including the crude fibers, such as cellulose on a dry solid basis.

**Fig. 1:**
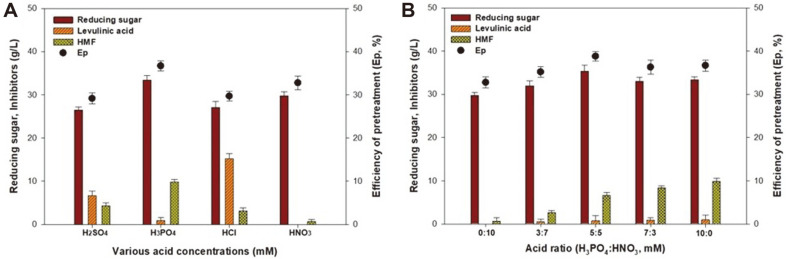
The effect of (**A**) various acid types and (**B**) acid ratios on the production of reducing sugar from *G. amansii* by HT acid hydrolysis. HT acid hydrolysis was carried out with a slurry concentration of 12% (w/v) and 360 mM of acid at 160°C for 15 min.

The HT acid hydrolysis pretreatment was carried out to determine the optimal acid type and combination ratios of H_2_SO_4_, H_3_PO_4_, HCl, and HNO_3_, as shown in Fig. 1. The effects of the various acid concentrations used were determined at a temperature of 160°C, thermal hydrolysis time of 15 min, and 12% (w/v) slurry as shown in Fig. 1A. Among the various acid treatments, H_3_PO_4_ produced the highest reducing sugar concentration with 33.4 g/l and *E*_p_ of 36.7%. Moreover, reducing sugar concentrations of 26.5 g/l (*E*_p_ = 29.1%), 27.0 g/l (*E*_p_ = 29.7%), and 29.8 g/l (*E*_p_ = 32.7%) were obtained when using H_2_SO_4_, HCl, and HNO_3_, respectively. On the other hand, the use of H_2_SO_4_, H_3_PO_4_, HCl, and HNO_3_ led to the production of 4.3, 9.8, 3.1, and 0.7 g/l of inhibitory compounds, such as 5-HMF. Furfural and 5-HMF are fermentation inhibitors and their formation indicated the need for separate detoxification steps for 2,3-BD fermentation [18]. Li *et al*. [19] reported on the performance of different acids combined with different salts used to convert glucose to 5-HMF. It was observed that H_2_SO_4_, H_3_PO_4_, and HCl produced a higher concentration of 5-HMF than HNO_3_. Thus, considering the conditions under which the maximum reducing sugar and minimum inhibitory compound concentrations were obtained, the combination of H_3_PO_4_ and HNO_3_ was selected as the suitable acid mixture for the HT acid hydrolysis pretreatment.

 The effects of the ratio between H_3_PO_4_ and HNO_3_ on the production of reducing sugar and inhibitors are shown in Fig. 1B. The results showed that a 5:5 ratio between H_3_PO_4_ and HNO_3_ produced the highest reducing sugar concentration, with 35.3 g/l and an *E*_p_ of 38.8%. Moreover, when we increased the proportion of H_3_PO_4_, we found that the concentration of reducing sugar and 5-HMF was not greater than that obtained when using a 5:5 ratio. Therefore, a 5:5 ratio between H_3_PO_4_ and HNO_3_ was selected as the optimal acid combination.

**Fig. 2:**
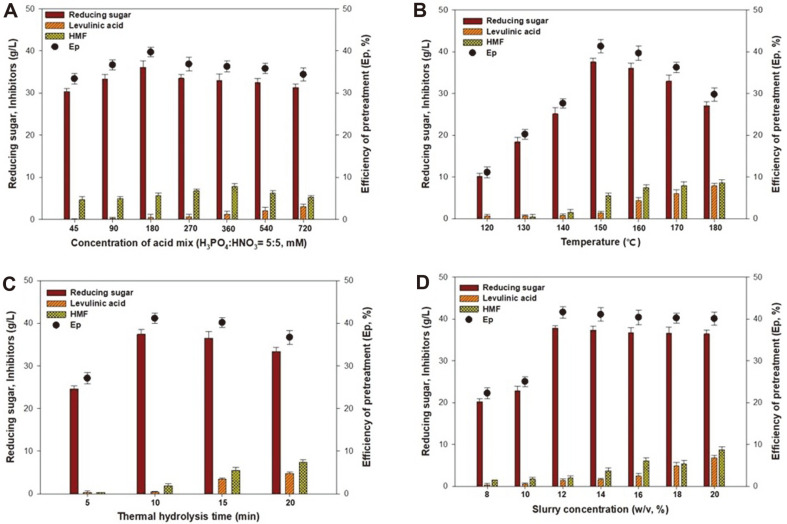
The evaluation of the HT acid hydrolysis conditions by changing the process parameters: (**A**) concentration of the acid mix, (**B**) reaction temperature, (**C**) thermal hydrolysis time, and (**D**) slurry concentration.

Next, we determined the optimal conditions for the main factors involved in the HT acid hydrolysis process, using H_3_PO_4_ and HNO_3_ (5:5 ratio) concentrations between 45–720 mM, temperatures ranging between 120–180°C, hydrolysis times of 5–20 min, and slurry concentrations between 8–20% (w/v) as shown in Fig. 2. The effects exerted by the varying H_3_PO_4_ and HNO_3_ (5:5 ratio) concentrations were determined at a temperature of 160°C, thermal hydrolysis time of 15 min, and 12% (w/v) slurry as shown in Fig. 2A. Our results showed that the reducing sugar concentration increased when increasing the concentration of the acid mixture to 180 mM. Therefore, the maximum reducing sugar concentration of 36.1 g/l and an *E*_p_ of 39.7% were obtained when using an acid mixture concentration of 180 mM. Further increases in concentration did not lead to significant increases in reducing sugar concentrations. Notably, reducing sugar production was reduced when the concentration was higher than 180 mM. Therefore, an acid mixture concentration of 180 mM was selected for HT acid hydrolysis pretreatment.

As shown in Fig. 2B, the temperature experiments were performed using 12% (w/v) slurry, 180 mM acid mixture, and a thermal hydrolysis time of 15 min. Increasing the temperature from 120 to 150°C resulted in an increase in the concentration of reducing sugars. The maximum reducing sugar concentration was obtained at 150°C, with 37.5 g/l and an *E*_p_ of 41.3%. However, increasing the temperature above 150°C resulted in a decrease in the concentration of reducing sugar, as well as an increase in the production of inhibitory compounds, such as 5-HMF (8.65 g/l) and levulinic acid (7.87 g/l) at 180°C. This indicates that excessive degradation of hexoses occurred at temperatures higher than 150°C. Similar glucose decomposition during acid-catalyzed hydrothermal hydrolysis of pretreated *Gelidium amansii* was reported by Jeong *et al*. [20]. Therefore, 150°C was chosen as the optimal reaction temperature.

The effects of different thermal hydrolysis times were determined when performing HT acid hydrolysis using of 12% (w/v) slurry, 180 mM of the acid mixture, and a reaction temperature of 150°C as shown in Fig. 2C. Our results showed that the reducing sugar concentration increased when the thermal hydrolysis time was increased to 10 min and then decreased with a further increase from 10 to 20 min. Similar results were obtained in other studies for the degradation of monosaccharides and the formation of inhibitory compounds by extended exposure to high temperature or a long thermal hydrolysis time [21]. Due to the reducing sugar concentrations and inhibitory effect of 5-HMF and levulinic acid, a 10 min thermal hydrolysis time was used in subsequent experiments.

The effects of using various slurry concentrations were determined at a temperature of 150°C, thermal hydrolysis time of 10 min, and an acid mixture concentration of 180 mM as shown in Fig. 2D. We found that the *E*_p_ when using a slurry concentration of 12% (w/v) was 41.6%, with a reducing sugar production of 37.8 g/l. Increases in the slurry concentration during HT acid hydrolysis beyond 12% (w/v) resulted in a decrease in the *E*_p_ from 41.6% to 40.0%. Therefore, a slurry concentration of 12% (w/v) was considered optimal for use in combination with the other parameters. These results indicate that the most effective HT acid hydrolysis conditions which prevent any damage to yeast fermentation were a slurry concentration of 12% (w/v), an acid mixture concentration of 180 mM, a reaction temperature of 150°C, and a reaction time of 10 min.

On the other hand, the opposite effect was observed for 5-HMF [22]. Previous studies have reported that 5-HMF was very useful not only as an intermediate for the production of biofuel, dimethylfuran (DMF) and other molecules, but also for the production of other important molecules such as levulinic acid, 2,5-furandicarboxylic acid (FDA), 2,5-diformylfuran (DFF), dihydroxymethylfuran, and 5-hydroxy-4-keto-2-pentenoic acid. Thus, considerable efforts have been made to transform carbohydrates into 5-HMF [23, 24].

### Enzymatic Saccharification

**Fig. 3:**
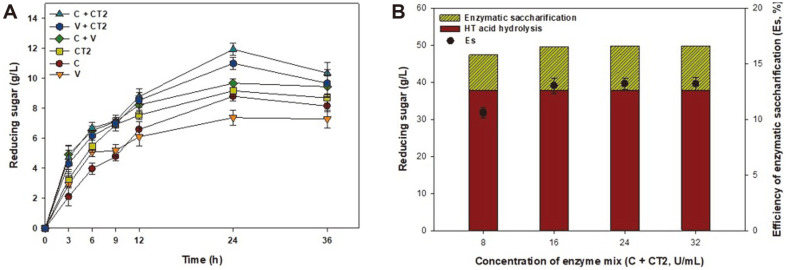
The effect of the (**A**) enzyme type and (**B**) enzyme dosage on the reducing sugar release of the *G. amansii* hydrolysate when using 12% (w/v) slurry after HT acid hydrolysis at pH 5.0, 40°C for 24 h. The initial reducing sugar concentration after HT acid hydrolysis pretreatment was 37.8 g/l, where C is Celluclast 1.5L, CT2 is CTec 2, V is Viscozyme L, and Es is efficiency of enzymatic saccharification.

The effects of single and mixed enzyme treatments using enzyme concentrations of 16 U/ml after HT acid hydrolysis were evaluated, as shown in Fig. 3. The initial reducing sugar concentration after HT acid hydrolysis pretreatment was 37.8 g/l. As shown in Fig. 3A, our results revealed that the optimal enzyme reaction time was 24 h and a further increase in reaction time to 36 h had no significant effect on reducing sugar production. A single treatment with Celluclast 1.5 L, Viscozyme L, or CTec2, led to an increase in reducing sugar concentration of 8.8 g/l (*E*_s_ = 9.7%), 7.4 g/l (*E*_s_ = 8.1%), and 9.2 g/l (*E*_s_ = 10.1%), respectively, as shown in Fig. 3A. However, treatment with a mixture of enzymes was shown to have a synergistic effect compared to the single enzyme treatments. Among the treatments, treatment with a mixture of Celluclast 1.5 L and CTec2 was preferable to the other single and mixed enzyme treatments. Amamou *et al*. [25] reported that a mix of enzymes exhibited higher degradation activity compared to single-enzyme treatments. Thus, treatment with a mixture between Celluclast 1.5 L and CTec2 was chosen for further experiments.

To determine the optimal dosage of the enzyme mix, enzymatic saccharification was carried out using various enzyme mixture activities (8–32 U/ml) at 50°C and 150 rpm for 24 h as shown in Fig. 3B. The optimum enzymatic activity was 16 U/ml and further increases in enzyme activity up to 32 U/ml showed no significant effect on reducing sugar concentration. Therefore, the maximum reducing sugar concentration and Es obtained were 12.0 g/l and 13.2% using a dose of 16 U/ml of the Celluclast 1.5 L and CTec2 mixture, respectively. Therefore, the enzyme mixture consisting of Celluclast 1.5 L and CTec2 at a concentration of 16 U/ml was selected for the enzymatic saccharification of the *G. amansii* hydrolysate. The efficiency of the pretreatment and enzymatic saccharification was 54.8%, with 49.8 g/l reducing sugar obtained from a total carbohydrate concentration of 90.96 g/l and 120 g dcw/L of the *G. amansii* slurry.

### 2,3-BD Fermentation by Engineered *S. cerevisiae* in *G. amansii* Hydrolysate

**Fig. 4:**
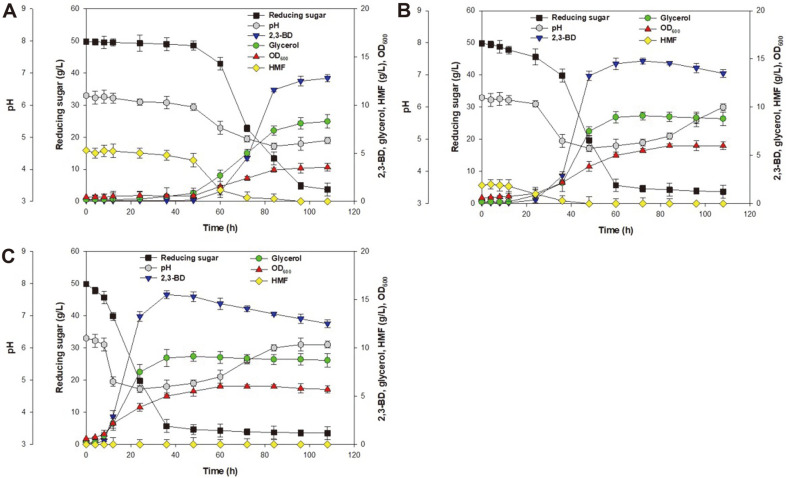
2,3-BD production from *G. amansii* by separate hydrolysis and fermentation (SHF) using engineered *S. cerevisiae* with (A) general thermal acid-hydrolyzed medium (12°C heat treatment for 60 min) with 5.3 g/l 5- HMF, (B) HT acid-hydrolyzed medium with 1.9 g/l 5-HMF, and (C) HT acid-hydrolyzed medium without 5- HMF using activated carbon.

The fermentation process was carried out by inoculating 6.8 g dcw/L of engineered *S. cerevisiae* into the HT acid-hydrolyzed and enzyme-hydrolyzed *G. amansii* slurry. As shown in Fig. 4, batch fermentation of engineered *S. cerevisiae* was carried out using a general thermal acid-hydrolyzed medium (121°C heat treatment for 60 min) with 5.3 g/l of 5-HMF (Fig. 4A), an HT acid-hydrolyzed medium with 1.9 g/l of 5-HMF (Fig. 4B), and an HT acid-hydrolyzed medium without 5-HMF using activated carbon (Fig. 4C).

As shown in Fig. 4A, the results for the fermentation process in the thermal acid-hydrolyzed medium with engineered *S. cerevisiae* showed a lag time of 48 h for 2,3-BD production due to the presence of 5-HMF. The 2,3-BD concentration and the cell growth were increased from 60 to 108 h when the 5-HMF concentration was decreased to near zero during the fermentation process. Similar results were obtained for ethanol production. The presence of 5-HMF in concentrations close to zero in the fermentation broth resulted in a rapid uptake of reducing sugar, cell growth, and ethanol production [26]. The engineered *S. cerevisiae* fermentation in the thermal acid-hydrolyzed medium produced 12.8 g/l 2,3-BD and a 2,3-BD yield (*Y*_BD_, g/g) of 0.26 at 108 h. As indicated by these results, the presence of an inhibitory compound influences not only the yeast strain, with respect to the sugar metabolic pathways, but also the formation of 2,3-BD during yeast fermentation. Jiang *et al*. [18] compared the yields of water-soluble products, reducing sugars, and furfurals (5-HMF and furfural) from *Jatropha* hulls. They reported that the furfural and 5-HMF present in the hydrolysates were inhibitors and removed them by charcoal adsorption during 2,3-BD fermentation. Thus, the presence of furfurals in the fermentation medium requires the separation of 5-HMF and furfural in detoxification steps.

As shown in Fig. 4B, we observed significant differences in the efficiency of the fermentation process when using the HT acid-hydrolyzed medium. The use of the HT acid-hydrolyzed medium with 1.9 g/l of 5-HMF led to a reduction in the lag time from 48 to 24 h when compared to using a general thermal acid-hydrolyzed medium. Moreover, the reducing sugar was consumed until 60 h into the fermentation process, with a resulting 2,3-BD concentration and 2,3-BD yield coefficient of 14.8 g/l and 0.30 at 72 h, respectively. These results indicate that the decreased 5-HMF concentration successfully improved the efficiency of the production of 2,3-BD via fermentation of reducing sugar. Thus, HT acid hydrolysis pretreatment is important to obtain reducing sugar with a low concentration of inhibitory compounds when the *G. amansii* hydrolysate is used as the carbon source.

To further improve the efficiency of the 2,3-BD production process, the activated carbon was added to the seaweed hydrolysates, as shown in Fig. 4C. A volume of 100 ml of the hydrolysate supplemented with 3% (w/v) activated carbon was placed in a shaking water bath at 100 rpm and 50°C for an adsorption time of 5 min. This resulted in 100% of the 5-HMF removal by activated carbon adsorption (data not shown) and a similar result was also obtained by a previous report [27]. Fig. 4C shows that after detoxification, the reducing sugar was consumed until 36 h and 3.5 g/l of reducing sugar remained in the fermentation medium. The engineered *S. cerevisiae* produced 2,3-BD at a concentration of 15.5 g/l and *Y*_BD_ of 0.31 during 36 h of fermentation. This indicates that the detoxification step can increase sugar utilization from seaweed hydrolysates, resulting in higher 2,3-BD yields in the fermentation broth. The 2,3-BD yield in this study was higher than that of the engineered *S. cerevisiae* BY4741, which has a *Y*_BD_ of 0.11 [28]. Thus, the use of activated carbon in *G. amansii* hydrolysates before fermentation has been shown to reduce the concentration of inhibitors effectively. This indicates that the engineered *S. cerevisiae* is a promising strain for increasing the 2,3-BD yield. Based on the obtained results, the evaluation of the HT acid hydrolysis process and the engineered *S. cerevisiae* strain could facilitate the efficient utilization of reducing sugar for the production of 2,3-BD from *G. amansii* hydrolysates.

In this study, HT acid hydrolysis was shown to produce significant reducing sugar concentrations and minimum inhibitory compounds. Therefore, 2,3-BD fermentation could be conducted without any damage to the sugar metabolism of the engineered *S. cerevisiae* strain. Among the three enzyme treatments investigated, a mixture of Celluclast 1.5 L and CTec2 showed preferable result to the other single and mixed enzyme treatments for enzymatic saccharification. Notably, the 2,3-BD concentration and *Y*_BD_ produced using the engineered *S. cerevisiae* strain was 14.8 g/l and 0.30 at 72 h, respectively. The fermentation profiles of the engineered *S. cerevisiae* provided a basis for the production of 2,3-BD via fermentation using the *G. amansii* hydrolysate as a substrate.
